# Involvement of Endolysosomes and Aurora Kinase A in the Regulation of Amyloid β Protein Levels in Neurons

**DOI:** 10.3390/ijms25116200

**Published:** 2024-06-04

**Authors:** Zahra Afghah, Nabab Khan, Gaurav Datta, Peter W. Halcrow, Jonathan D. Geiger, Xuesong Chen

**Affiliations:** Department of Biomedical Sciences, University of North Dakota School of Medicine and Health Sciences, Grand Forks, ND 58203, USA; zahra.afghah@ndus.edu (Z.A.); nabab.khan@yale.edu (N.K.); gaurav.datta@ndus.edu (G.D.); peter.halcrow@und.edu (P.W.H.); jonathan.geiger@und.edu (J.D.G.)

**Keywords:** AURKA, endolysosomes, amyloid β, BACE-1, cathepsin D

## Abstract

Aurora kinase A (AURKA) is a serine/threonine-protein kinase that regulates microtubule organization during neuron migration and neurite formation. Decreased activity of AURKA was found in Alzheimer’s disease (AD) brain samples, but little is known about the role of AURKA in AD pathogenesis. Here, we demonstrate that AURKA is expressed in primary cultured rat neurons, neurons from adult mouse brains, and neurons in postmortem human AD brains. AURKA phosphorylation, which positively correlates with its activity, is reduced in human AD brains. In SH-SY5Y cells, pharmacological activation of AURKA increased AURKA phosphorylation, acidified endolysosomes, decreased the activity of amyloid beta protein (Aβ) generating enzyme β-site amyloid precursor protein cleaving enzyme (BACE-1), increased the activity of the Aβ degrading enzyme cathepsin D, and decreased the intracellular and secreted levels of Aβ. Conversely, pharmacological inhibition of AURKA decreased AURKA phosphorylation, de-acidified endolysosomes, decreased the activity of cathepsin D, and increased intracellular and secreted levels of Aβ. Thus, reduced AURKA activity in AD may contribute to the development of intraneuronal accumulations of Aβ and extracellular amyloid plaque formation.

## 1. Introduction

Alzheimer’s disease (AD) is the most common form of dementia, and there are limited treatments for this progressive age-related neurodegenerative disease. AD is characterized clinically by progressive cognitive decline and memory impairment and pathologically by the presence of extracellular senile plaques composed of amyloid β (Aβ) protein, intracellular neurofibrillary tangles composed of hyperphosphorylated tau, and synaptic and neuronal loss [1]. Endolysosome dysfunction, an early pathological feature of AD [2,3], contributes to the development of pathological features commonly implicated in AD pathogenesis [4].

Endolysosome acidity is maintained mainly by vacuolar H^+^-ATPases (v-ATPases) [5] and the luminal acidic pH controls the activity of nearly 60 different pH-sensitive hydrolytic enzymes [6]. The enzymes responsible for the amyloidogenic processing of amyloid β precursor protein (AβPP) including β- and γ-secretases are present in endolysosomes, and their activities are pH-dependent [7,8]. Further, Aβ is degraded by the lysosome enzymes cathepsin D and B [9,10]. Non-degraded Aβ can accumulate inside neurons as intraneuronal Aβ or it can be released to the extracellular space via exocytosis where it can contribute to the formation of senile plaques. Endolysosome de-acidification can increase intra- and extracellular levels of Aβ [11,12,13]. On the other hand, endolysosome acidification can decrease the production of Aβ [12,14,15].

Aurora kinase A (AURKA) is a serine/threonine-protein kinase that regulates microtubule dynamics, influencing microtubule stability, organization, and turnover [16]. AURKA has been shown to regulate neuronal function such as microtubule organization, neuronal migration, and synaptic plasticity [17,18,19], and decreased AURKA activity was found in postmortem AD brains [20]. However, virtually nothing is known about the expression of AURKA in the brain and the extent to which AURKA contributes to AD pathogenesis. Because AURKA modulates the activity of v-ATPase, the proton pump maintains the acidic pH of endolysosomes, in non-neuronal cells [21], and because endolysosome de-acidification can increase intra- and extracellular levels of Aβ [11,12,13], we tested the hypothesis that AURKA through endolysosome-mediated actions affects Aβ generation in neurons. Our findings that AURKA was expressed in neurons and that activating AURKA acidified endolysosomes and decreased Aβ levels whereas inhibiting AURKA de-acidified endolysosomes and increased Aβ levels highlight the role of AURKA in modulating endolysosome function and Aβ generation.

## 2. Results

### 2.1. AURKA Is Expressed in Neurons: AURKA Was Highly Present in Primary Cultured Rat Cortical Neurons (Figure 1A)

**Figure 1:**
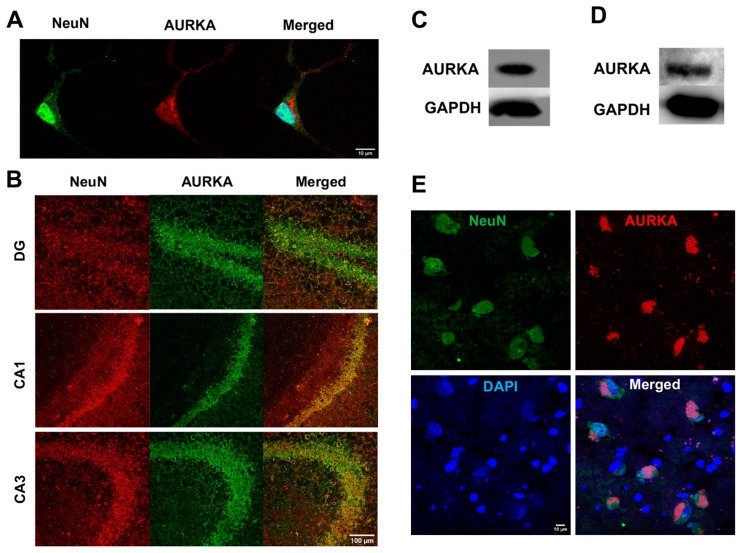
AURKA is expressed in neurons. (**A**) AURKA (red) was expressed in primary cultured rat cortical neurons (NeuN, green). Nuclei were stained with DAPI. (**B**) AURKA (green) was expressed in neurons NeuN (red) in the dentate gyrus (DG), CA1, and CA3 subregions of the hippocampus in the 8-month-old adult C57BL/6J mouse brains. (**C**) Immunoblots showed that AURKA was expressed in adult mouse brains. (**D**) Immunoblots showed that AURKA was expressed in the adult rat brain. (**E**) AURKA (red) was expressed in neurons (NeuN, green) in fresh-frozen postmortem human brain hippocampal samples.

In the hippocampus of adult mouse brains, AURKA was highly expressed in neurons of the dentate gyrus (DG) as well as the CA1 and CA3 subregions of hippocampus (Figure 1B). AURKA, as determined with immunoblotting, was present in adult C57BL/6J mouse brains (Figure 1C) and adult Sprague-Dawley rat brains (Figure 1D). AURKA was also expressed in neurons labeled with NeuN in postmortem human brains (Figure 1E).

### 2.2. Phosphorylated AURKA Is Reduced in Human AD Brain

**Figure 2:**
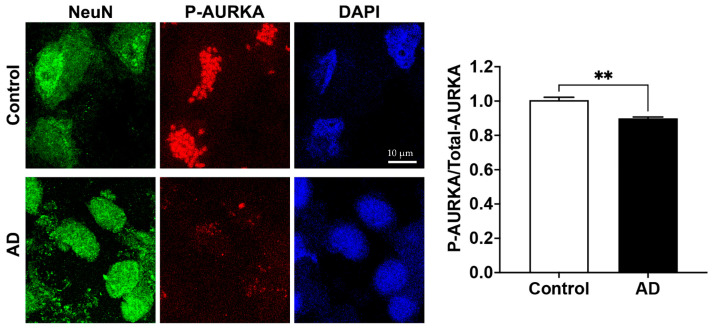
Phosphorylated AURKA (active form) is reduced in AD human brains. Confocal images showed that AURKA (red) phosphorylation was significantly decreased in neurons labeled with NeuN (green) in the hippocampus of the human AD brain compared to the control brain (*n* = 3 repeats using different slides, ** *p* < 0.01).

The relative fluorescent units (RFU) for both phosphorylated AURKA and total AURKA in neurons (NeuN) were decreased in the AD human brain when compared to the control. The RFU of phosphorylated AURKA was 78.91 ± 4.52 in the control vs. 51.52 ± 1.86 in AD (*n* = 3, *p* < 0.001); the RFU of total AURKA was 78.62 ± 3.77 in the control vs. 57.31 ± 0.88 in AD (*n* = 3, *p* < 0.001). When quantifying P-AURKA normalized to the total AURKA and then related to the control, we found that phosphorylated AURKA levels were significantly decreased in neurons (NeuN) from postmortem human AD brains compared with the control brain (Figure 2). However, we only managed to obtain frozen hippocampal brain tissue sections (5–10 µm thickness) mounted on slides from BioChain, and these brain tissues samples are from one female AD donor and one age-matched female control donor. Furthermore, no clinical data on memory and cognitive function are available. This represents a significant limitation of our study.

### 2.3. AURKA Affects Endolysosome pH

**Figure 3:**
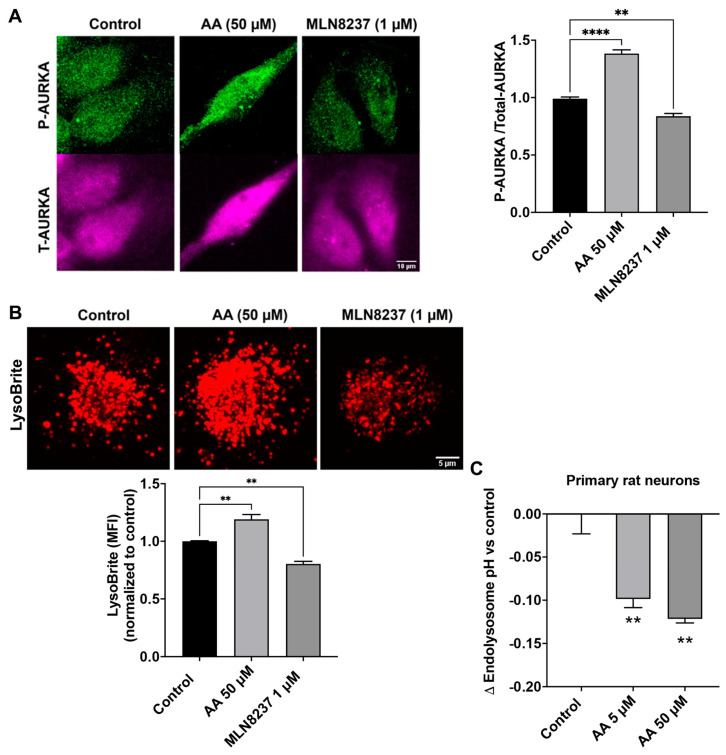
AURKA affects endolysosome pH. (**A**) In SH-SY5Y cells, AURKA activator anacardic acid (AA 50 µM for 4 h) significantly increased phosphorylation of AURKA, whereas AURKA inhibitor (MLN8237 1 µM for 4 h) significantly decreased phosphorylation of AURKA compared to the DMSO control group. (*n* = 3 repeats, ** *p* < 0.01, **** *p* < 0.0001). (**B**) AURKA activator anacardic acid (50 µM for 4 h) increased LysoBrite fluorescence, whereas AURKA inhibitor MLN8237 (1 µM for 4 h) decreased LysoBrite fluorescence in SH-SY5Y cells (*n* = 3 repeats, ** *p* < 0.01). (**C**) Activating AURKA with 5 µM (*n* = 3 repeats, ** *p* < 0.01) and 50 µM (*n* = 3 repeats, ** *p* < 0.01) of anacardic acid for 4 h significantly decreased endolysosome pH in primary cultured neurons.

Phosphorylated AURKA enhances v-ATPase activity—a finding noted in non-neuronal cells [21]. Here, we determined the extent to which pharmacological activation or inhibition of AURKA affected levels of phosphorylated AURKA and endolysosome pH in SH-SY5Y cells. The specific AURKA activator anacardic acid (50 µM for 4 h) significantly increased AURKA phosphorylation, whereas the specific inhibitor for AURKA MLN8237 (1 µM for 4 h), significantly decreased phosphorylated levels of AURKA by 17% when compared to the control (Figure 3A). Next, we assessed the effect of activating AURKA or inhibiting AURKA on endolysosome pH with LysoBrite. The accumulation of LysoBrite in endolysosomes depends on the pH gradient, and thus changes in the fluorescent intensity of LysoBrite can assess changes in endolysosome pH; increased fluorescent intensity of LysoBrite indicates endolysosome acidification, whereas decreased fluorescent intensity of LysoBrite indicates endolysosome de-acidification. We found that activating AURKA with anacardic acid (50 µM for 4 h) increased LysoBrite fluorescence; an indication of endolysosome acidification (Figure 3B). Inhibiting AURKA with MLN8237 (1 µM for 4 h) decreased LysoBrite fluorescence—an indication of endolysosome de-acidification (Figure 3B). It should be noted that changes in endolysosome population can also affect the fluorescent intensity of LysoBrite, and we assessed the effect of AURKA activation and inhibition on the population and distribution of LAMP1-labeled endolysosomes in a study assessing mitosis that is unrelated to this manuscript. We observed that neither activating nor inhibiting AURKA activity affects the population of endolysosomes, but inhibiting AURKA does affect the distribution of endolysosomes with endolysosomes being clustered together, which correlates with impaired mitosis. Such a change in endolysosome distribution may result from the combined effect of microtubule modulation and endolysosome de-acidification resulting from AURKA inhibition, given that both microtubule modulation and endolysosome de-acidification could affect the mobility and dynamics of endolysosomes. Thus, our findings suggest that activating AURKA acidifies endolysosomes, whereas inhibiting AURKA de-acidifies endolysosomes. Furthermore, using a ratiometric measurement of endolysosome pH in primary rat cortical neurons, we demonstrated that activation of AURKA with anacardic acid (5 and 50 µM) significantly decreased endolysosome pH (Figure 3C). However, inhibiting AURKA with MLN8237 (1 µM) did not lead to statistically significant changes in endolysosome pH.

### 2.4. AURKA Affects Aβ Generation

**Figure 4:**
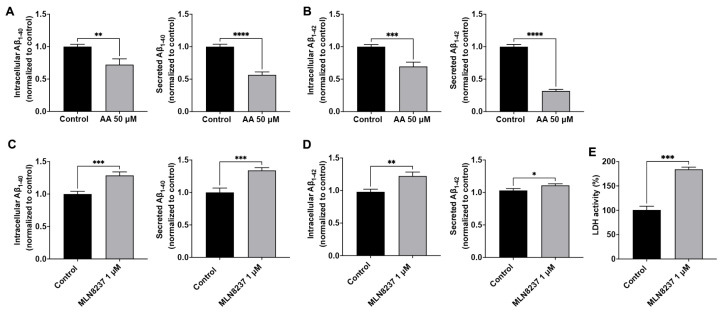
AURKA affects Aβ. Activating AURKA with anacardic acid (AA, 50 μM for 2 days) significantly decreased intracellular and secreted levels of Aβ_1-40_ (**A**) and Aβ_1-42_ (**B**) in SH-SY5Y cells (*n* = 4 repeats, ** *p* < 0.01, *** *p* < 0.001, **** *p* < 0.0001). Inhibiting AURKA with MLN8237 (1 μM for 2 days) significantly increased intracellular and secreted levels of Aβ_1-40_ (**C**) and Aβ_1-42_ (**D**) in SH-SY5Y cells (*n* = 4 repeats, * *p* < 0.05, ** *p* < 0.01, *** *p* < 0.001). (**E**) MLN8237 (1 µM for 2 days) significantly increased released LDH activity (*n* = 3 repeats, *** *p* < 0.001).

We reported previously that endolysosome pH regulated Aβ levels [12,14,15], and here we determined the extent to which modulating AURKA activity affects Aβ levels. The Aβ peptide sequence of AβPP in rodents is different from that in humans, rodents do not spontaneously develop amyloid deposits, and the amount of Aβ secreted from primary rodent neurons is very low and often undetectable. As such, rodent neuronal cells (Neuro-2a, for example) overexpressing human AβPP are usually needed for studying Aβ processing and generation. Instead of using rodent neuronal cells, we chose to use human SH-SY5Y cells that overexpress AβPP695, the major neuronal AβPP isoform in humans, for studying the effect of AURKA on Aβ generation. In control (DMSO-treated) SH-SY5Y over-expressing AβPP cells, the maximum secreted Aβ_1-40_ was 1300 pmol/mg of protein, and the maximum secreted Aβ_1-42_ was 400 pmol/mg of protein. Activating AURKA with anacardic acid (50 µM for 2 days) significantly decreased intracellular levels of Aβ_1-40_ and secreted levels of Aβ_1-40_ (Figure 4A), as well as intracellular levels of Aβ_1-42_ and secreted levels of Aβ_1-42_ (Figure 4B). Using an LDH release assay, we demonstrated that anacardic acid (50 µM for 2 days) did not significantly increase LDH release. AURKA inhibition with MLN8237 (1 µM for 2 days) significantly increased intracellular levels of Aβ_1-40_ and secreted levels of Aβ_1-40_ (Figure 4C), as well as intracellular levels of Aβ_1-42_ and secreted levels of Aβ_1-42_ (Figure 4D). Using the LDH release assay, we demonstrated that MLN8237 (1 µM for 2 days) significantly increased released LDH activity (Figure 4E).

### 2.5. AURKA Activity Effects on BACE-1 and Cathepsin D

**Figure 5:**
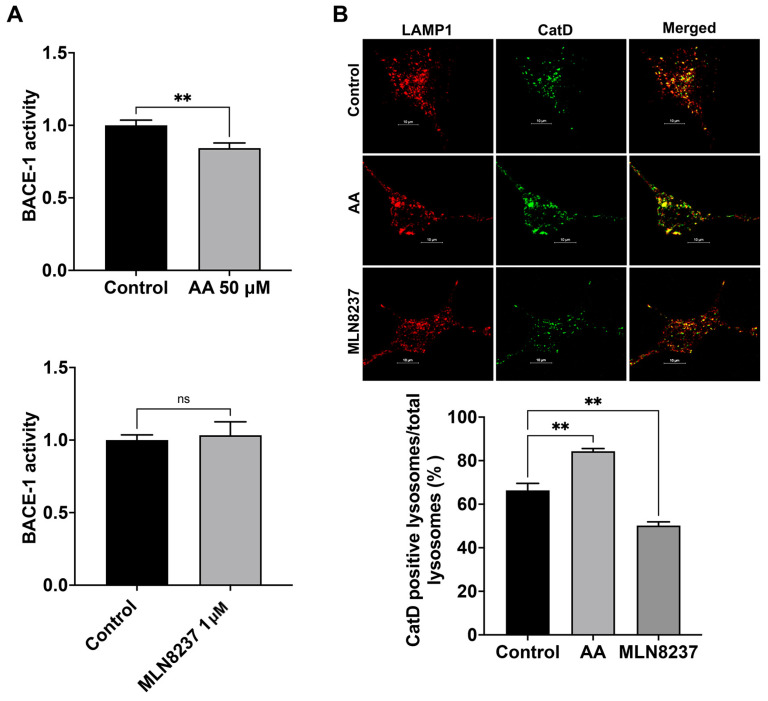
AURKA affects BACE-1 and cathepsin D activity. (**A**) AURKA activator anacardic acid (AA, 50 μM for 2 days), but not AURKA inhibitor MLN8237 (1 μM for 2 days), decreased BACE-1 activity in SH-SY5Y cells (*n* = 3 repeats, ns: not significant, ** *p* < 0.01). (**B**) AURKA activator anacardic acid (AA, 50 μM for 2 days) significantly increased the percentage of cathepsin D-positive endolysosomes identified with LAMP1. AURKA inhibitor MLN8237 (1 μM for 2 days) significantly decreased the percentage of cathepsin D-positive endolysosomes identified with LAMP1 (*n* = 3 repeats, ** *p* < 0.01).

We determined here the extent to which AURKA activity affects the activity of BACE-1, a critical and rate-limiting enzyme in the production of Aβ [22]. We found that activating AURKA with anacardic acid (50 µM for 2 days) significantly reduced BACE-1 activity (Figure 5A). Inhibiting AURKA with MLN8237 (1 µM for 2 days) did not significantly change the activity of BACE-1 (Figure 5A). Because Aβ generated in endosomes can be degraded by cathepsin D [23], which exhibits an optimum pH of 3–4.5 and thus is more active in more acidic late endosomes and lysosomes [9,24,25,26,27], we assessed the extent to which AURKA activation and inhibition affect total protein levels of cathepsin D with immunoblotting. We observed that neither AURKA activation nor inhibition altered protein levels of cathepsin after 4 hr treatment. However, following 2-day treatment, AURKA activation with AA (50 μM) decreased total protein levels of cathepsin D by 19%, but AURKA inhibition with MLN8237 (10 μM) drastically decreased total protein levels of cathepsin D by 48%. Such changes in total protein levels of cathepsin D may result from a combined effect of synthesis, degradation, and secretion of cathepsin D. Because AURKA plays a critical role in modulating microtubule dynamics, influencing microtubule stability, organization, and turnover [16], AURKA could affect vesicle trafficking and the transport of lysosomal enzymes from Golgi to lysosomes, we chose to determine the extent to which AURKA affects cathepsin D enzyme activity using an assay based on BODIPY-FL Pepstatin A. As a cathepsin D inhibitor, BODIPY-FL Pepstatin A binds specifically to the active form of cathepsin D, and such a binding turns on its fluorescence. Thus, the fluorescent intensity of BODIPY-FL Pepstatin A is not only used as a marker of cathepsin D presence but is also used for assessing cathepsin D activity [28,29]. An increased percentage of active cathepsin D positive endolysosomes indicates enhanced cathepsin D activity. We found that activating AURKA significantly increased the percentage of active cathepsin D-positive endolysosomes (Figure 5B), whereas inhibiting AURKA with MLN8237 significantly decreased the percentage of active cathepsin D-positive endolysosomes (Figure 5B).

## 3. Discussion

We report here that (1) AURKA phosphorylation was decreased in the human AD brain, (2) pharmacological activation of AURKA increased AURKA phosphorylation, acidified endolysosomes, decreased the activity of BACE-1, increased the activity of cathepsin D, and decreased intracellular and secreted levels of Aβ_1-40_ and Aβ_1-42_, and (3) pharmacological inhibition of AURKA decreased AURKA phosphorylation, de-acidified endolysosomes, decreased the activity of cathepsin D, and increased intracellular and secreted levels of Aβ_1-40_ and Aβ_1-42_.

AURKA is a serine/threonine kinase that regulates mitosis and cell division [30,31], controls mitochondrial dynamics and energy production [32,33], and promotes glycolytic metabolism [34]. AURKA has also been implicated in regulating post-mitotic neuronal functions including microtubule organization during neuronal migration and neurite formation [17,18,19]. AURKA is expressed in primary cultured neurons [19] and we demonstrated here that AURKA was expressed in mature and immature neurons. Thus, AURKA might help regulate brain development and the pathogenesis of age-related neurological disorders. AURKA was first implicated in the pathogenesis of AD with findings that AURKA activity was decreased in the human AD brain [20] and we showed here that AURKA activity (as indicated by its phosphorylation status) was decreased in the human AD brain.

Endolysosome dysfunction is an early pathological feature of AD [2,3] and contributes to the development of Aβ pathology [4]. We demonstrated that activating AURKA acidified endolysosomes and inhibiting AURKA de-acidified endolysosomes, possibly through actions on Ser-175 phosphorylation sites on the v-ATPase subunit A [21]. The acidic luminal pH of endolysosomes helps regulate the activities of various endolysosome-resident hydrolases—enzymes that catalyze the degradation of macromolecules and regulate waste accumulation in neurons. Amyloidogenic processing of AβPP and the degradation of Aβ are controlled by BACE-1, γ-secretases, and cathepsins—enzymes with optimal activity in acidic environments [7,8,9,10]. Thus, endolysosome de-acidification decreases the activity levels of enzymes that catalyze Aβ degradation and enhance BACE-1 activity, whereas endolysosome acidification can reduce Aβ levels [12,14,15].

Intraneuronal accumulation of especially oligomeric Aβ is neurotoxic and precedes Aβ extracellular plaque formation [35]. We demonstrated here that AURKA activation and endolysosome acidification decreased both intracellular and secreted levels of Aβ, whereas inhibiting AURKA and endolysosome de-acidification increased intracellular and secreted levels of Aβ. This may be due to pH-dependent changes in BACE-1 and γ-secretase enzyme activity and/or enzyme degradation. Indeed, AURKA activation, but not inhibition, affected levels of BACE-1 activity possibly because of the involvement of other factors including cathepsins. Indeed, we found that activating AURKA acidified endolysosomes and increased the activity of cathepsin D, whereas inhibiting AURKA de-acidified endolysosomes and decreased the activity of cathepsin D. Thus, AURKA activity appears to affect Aβ levels via its actions on endolysosome pH and the activity of enzymes that catalyze Aβ metabolism. However, AURKA plays a critical role in modulating microtubule dynamics, influencing microtubule stability, organization, and turnover [16]. As such, AURKA could affect vesicle trafficking and the transport of lysosomal enzymes from Golgi to lysosomes. AURKA inhibition may block the transport of cathepsin D from Golgi to endolysosomes, and such an effect may work together with decreased cathepsin D activity, resulting from endolysosome de-acidification, to impair the degradation of Aβ, thus increasing Aβ levels. Furthermore, cytotoxic effect induced by AURKA inhibitor MLN8237 could also contribute to its effect in increasing Aβ. Here, only a pharmacological activator and inhibitor of AURKA are used. As specificity represents an issue for pharmacological reagents, future studies assessing the effect of AURKA knockout on Aβ are warranted.

Together, our findings demonstrate a novel role of AURKA in modulating endolysosome function and Aβ metabolism. Our findings suggest that reduced AURKA activity in AD could contribute to developing intraneuronal accumulation of Aβ and extracellular amyloid plaque formation and that activating AURKA may represent a therapeutic strategy against AD.

## 4. Materials and Methods

Cell cultures: Primary cultures of cortical neurons were prepared from Sprague-Dawley rats. Pregnant rats were euthanized by asphyxiation with CO_2_ followed by decapitation at embryonic day 18. After the fetuses were removed and decapitated, meninges-free cerebral cortices were isolated, trypsinized (Corning, NY, USA), and plated in culture dishes. Neurons were grown in the Neurobasal^TM^ medium (Gibco, Grand Island, NY, USA) with L-glutamine, penicillin/streptomycin, and B27 supplement and maintained in an incubator at 37 °C and 5% CO_2_ for 7–14 days. Typically, the purity of the neuronal cultures was greater than 95%. SH-SY5Y cells overexpressing AβPP695, the major neuronal AβPP isoform in humans, were supplied by Dr. Norman Haughey (John Hopkins University). This overexpression leads to an approximate 50% increase in the expression of full-length AβPP (as measured by immunoblotting) in these cells. SH-SY5Y cells were cultured in Dulbecco’s modified Eagle’s medium (Gibco) supplemented with 10% fetal bovine serum, and 1% penicillin/streptomycin in a humidified incubator with 5% CO_2_ at 37 °C.

Reagents: Human brain samples (frozen hippocampal brain tissue sections (5–10 µm thickness) were purchased from BioChain Institute Inc., Newark, CA, USA (#: T1234035 and #: T1236052Alz). Primary antibodies: Mouse anti-NeuN (Abcam ab104224), rabbit anti-AURKA (Abcam ab61114), rabbit anti-phospho-AURKA (ThermoFisher 44-1210G), mouse anti-AURKA (Abcam ab13824), mouse anti-GAPDH (Abcam ab8245), rabbit anti-BACE1 (Abcam ab108394) were obtained from Abcam, Cambridge, UK. Secondary antibodies: Alexa 594 goat anti-rabbit (111-587-003), FITC goat anti-rabbit (111-095-003), Alexa 594 goat anti-mouse (115-586-006), and FITC goat anti-mouse (115-095-003) were obtained from Jackson ImmunoResearch, West Grove, PA, USA, Alexa 647 goat anti-mouse far-red (ab150115) was obtained from Abcam; goat anti-rabbit (926-32211) and goat anti-mouse (926-68070) were obtained from Licor, Lincoln, NE, USA.

Immunostaining: Cells were fixed with 4% paraformaldehyde for 20 min, followed by permeabilizing with cold methanol (−20 °C) for 10 min. Following blocking with 5% goat serum and incubation overnight at 4 °C with primary antibodies, cells were incubated for an hour at room temperature with corresponding fluorescence-conjugated secondary antibodies. Images were captured using an LSM800 laser-scanning microscope system (Zeiss, Oberkochen, Germany) and were analyzed using ImageJ software (Version: 2.0.0-rc-68/1.52e).

Immunoblotting: Cells were lysed with RIPA buffer (ThermoFisher, Waltham, MA USA) plus Halt™ Protease and Phosphatase Inhibitor Cocktail (ThermoFisher). Cell lysates were centrifuged at 14,000× *g* for 10 min at 4 °C, supernatants were collected, and protein concentrations were determined using the Bradford protein assay (Bio-Rad, Hercules, CA, USA). Proteins (30 μg) were separated by SDS-PAGE (12% gel) and transferred to polyvinylidene difluoride (PVDF) membranes (Invitrogen, Carlsbad, CA, USA). The PVDF membranes were incubated overnight with primary antibodies, and blots were incubated with secondary antibodies for one hour, visualized, and analyzed using the LI-COR Odyssey Fc Imaging System, Lincoln, NE, USA.

Endolysosome pH assessment with LysoBrite: Endolysosomes were stained with LysoBrite^TM^ Red (22645, AAT Bioquest, Pleasanton, CA, USA), which selectively accumulates inside endolysosomes via the pH gradient. Cells were treated for 3.5 h with either the 50 μM anacardic acid (Abcam), 1 μM MLN8237 (AdooQ Bioscience, Irvine, CA, USA), or 0.1% DMSO as the vehicle control. Following treatment, cells were incubated with LysoBrite^TM^ (1:500) in media for 30 min at 37 °C. Following washing with PBS, live cell images were captured using confocal microscopy (Zeiss LSM800). Images were analyzed using ImageJ software (NIH).

Ratiometric endolysosome pH measurement: Endolysosome pH was measured using a dual excitation ratiometric pH indicator dye LysoSensor (Yellow/Blue DND-160, Invitrogen). Cells treated with either anacardic acid (50 µM) or DMSO (0.1%) as a vehicle control for 4 h and were incubated with 2 µM of DND-160 for 5 min at 37 °C. The light emitted at 520 nm in response to excitation at 340 nm and 380 nm for 20 ms was measured every 5 s for up to 20 min using our filter-based imaging system (Zeiss Axiovert 200M, Germany). The ratios of light excited at 340/380 nm and emitted at 520 nm were converted to pH using a calibration curve as previously described [12,14,15].

Aβ measurement with ELISA: Cells were treated with either anacardic acid (50 μM), MLN8237 (1 μM), or DMSO (0.1%) for 2 days. Following treatments, intracellular and secreted levels of Aβ were measured using Human/Rat β Amyloid (40) and Human/Rat β Amyloid (42) ELISA kits from Wako according to the manufacturer’s protocols. For secreted levels of Aβ, supernatants were collected, centrifuged at 14,000× *g* for 20 min at 4 °C and diluted with BSAT-DPBS reaction buffer supplemented with 1X protease and phosphatase inhibitor cocktail. Aβ levels were normalized to the total protein content of samples using a Bradford assay (Bio-Rad).

Cell toxicity assay: Cell toxicity was quantitatively assessed by the measurement of lactate dehydrogenase (LDH) released from damaged or destroyed cells into the extracellular fluid (#88953, ThermoFisher). Cells were treated with anacardic acid (50 μM), MLN8237 (1 μM) for 48 h. DMSO was used as a vehicle control, and a positive control supplied by Pierce was used. Following treatment, an aliquot (50 µL) of bathing media was combined with NADH and pyruvate solutions. LDH activity is proportional to the rate of pyruvate loss, which was assayed by absorbance change using a BioTek Synergy H1 multimode reader (Agilent Technologies, Inc., Santa Clara, CA, USA). Data were expressed as percentages of the positive control.

BACE-1 activity assay: β-secretase (BACE-1) activity was determined using a fluorometric assay (MAK237, MilliporeSigma, Burlington, MA, USA) that uses a secretase-specific peptide conjugated to fluorescent reporter molecules. Cleavage of EDANS and DABCYL catalyzed by BACE-1 releases fluorescence measured using a fluorescence microplate reader (excitation 345 nm and emission 500 nm). Cells were treated with either anacardic acid (50 μM), MLN8237 (1 μM) or DMSO (0.1%) for 2 days. Following treatments, cells were lysed in ice-cold extraction buffer, centrifuged at 14,000× *g* for 10 min, and supernatants were collected, and protein concentrations were measured. The BACE-1 activity was tested in the presence and absence of the BACE-1 inhibitor provided with the kit. The levels of BACE-1 activity, which are proportional to the level of fluorescence intensity, were expressed as normalized data vs. the control.

Cathepsin D staining: Activity levels of cathepsin D were measured using an assay based on BODIPY-FL Pepstatin A (P12271, ThermoFisher). Cells were transduced with CellLight^®^ Lysosomes-RFP BacMam 2.0 (ThermoFisher, Waltham, MA USA) and treated with either anacardic acid (50 μM), MLN8237 (10 μM) or DMSO (0.1%) for 24 h. Active cathepsin D was labeled with BODIPY-FL Pepstatin A (1 μM, 30 min), and cells were visualized by imaging under a Zeiss LSM800 confocal microscope. The ratio of cathepsin D-positive endolysosomes using BODIPY-FL Pepstatin A vs. total endolysosomes using Lysosomes-RFP were analyzed using Imaris 9.6 software.

Statistical analysis: All data were presented as means ± standard error of the mean (SEM). The statistical significance between the two groups was analyzed using Student’s *t*-test. The statistical significance among multiple groups was determined using one-way ANOVA plus Tukey’s post hoc test. A value of *p* < 0.05 was considered to be statistically significant.

## Data Availability

The original contributions presented in the study are included in the article, further inquiries can be directed to the corresponding author/s.
